# Supplementary material for: Causal Relationship between Aging and Anorexia Nervosa: A White-Matter-Microstructure-Mediated Mendelian Randomization Analysis
Source: Biomedicines. 2024 Aug 16;12(8):1874. doi: 10.3390/biomedicines12081874 (PMC11351342; doi:10.3390/biomedicines12081874)
Supplement: Supplementary file 1 [file biomedicines-12-01874-s001.zip › Supplementary Figure/Figure S4/Singe SNP plot of MR analysis for TL to ubm-b-1488.pdf]

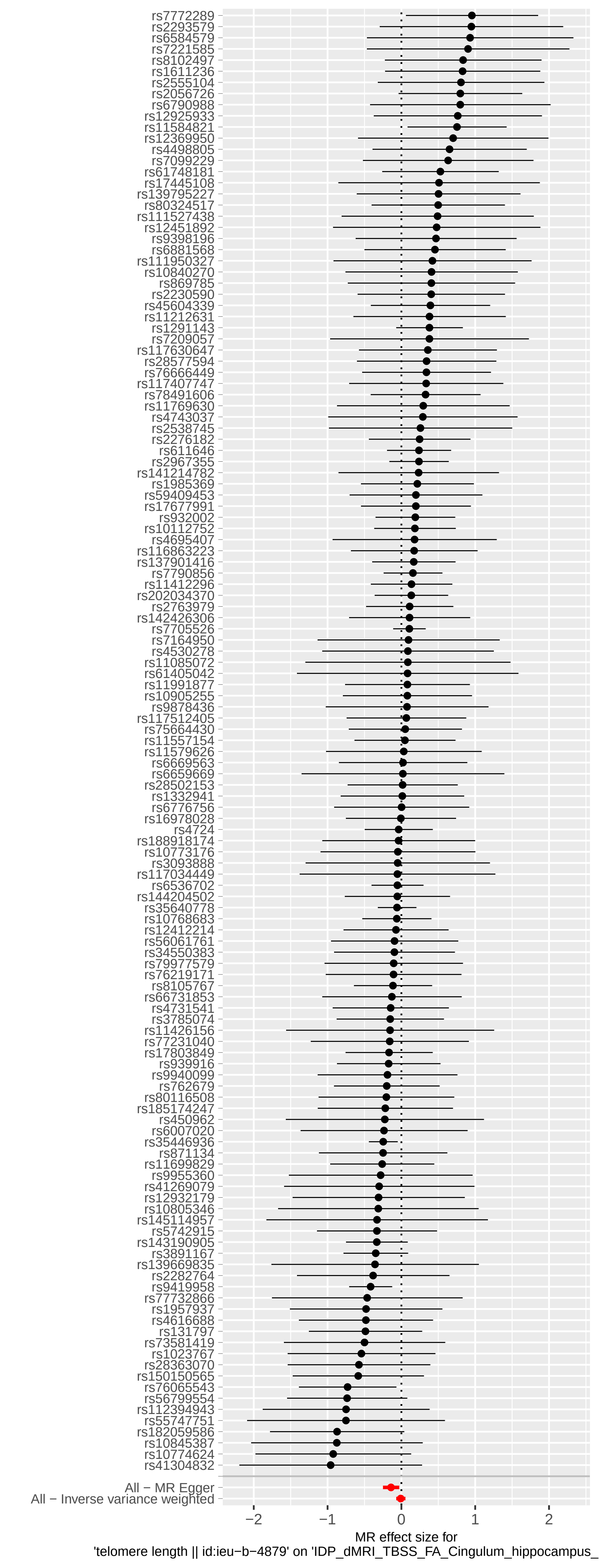

rs7772289
rs2293579
rs6584579
rs7221585
rs8102497
rs1611236
rs2555104
rs2056726
rs6790988
rs12925933
rs11584821
rs12369950
rs4498805
rs7099229
rs61748181
rs17445108
rs139795227
rs80324517
rs111527438
rs12451892
rs9398196
rs6881568
rs111950327
rs10840270
rs869785
rs2230590
rs45604339
rs11212631
rs1291143
rs7209057
rs117630647
rs28577594
rs76666449
rs117407747
rs78491606
rs11769630
rs4743037
rs2538745
rs2276182
rs611646
rs2967355
rs141214782
rs1985369
rs59409453
rs17677991
rs932002
rs10112752
rs4695407
rs116863223
rs137901416
rs7790856
rs11412296
rs202034370
rs2763979
rs142426306
rs7705526
rs7164950
rs4530278
rs11085072
rs61405042
rs11991877
rs10905255
rs9878436
rs117512405
rs75664430
rs11557154
rs11579626
rs6669563
rs6659669
rs28502153
rs1332941
rs6776756
rs16978028
rs4724
rs188918174
rs10773176
rs3093888
rs117034449
rs6536702
rs144204502
rs35640778
rs10768683
rs12412214
rs56061761
rs34550383
rs79977579
rs76219171
rs8105767
rs66731853
rs4731541
rs3785074
rs11426156
rs77231040
rs17803849
rs939916
rs9940099
rs762679
rs80116508
rs185174247
rs450962
rs6007020
rs35446936
rs871134
rs11699829
rs9955360
rs41269079
rs12932179
rs10805346
rs145114957
rs5742915
rs143190905
rs3891167
rs139669835
rs2282764
rs9419958
rs77732866
rs1957937
rs4616688
rs131797
rs73581419
rs1023767
rs28363070
rs150150565
rs76065543
rs56799554
rs112394943
rs55747751
rs182059586
rs10845387
rs10774624
rs41304832
All − MR Egger
All − Inverse variance weighted
−2
−1
0
1
2
MR effect size for
'telomere length || id:ieu−b−4879' on 'IDP_dMRI_TBSS_FA_Cingulum_hippocampus_
